# Intravenous thrombolysis in CADASIL: report of two cases and a systematic review

**DOI:** 10.1007/s10072-022-06449-2

**Published:** 2022-10-18

**Authors:** Francesca Pescini, Sara Torricelli, Martina Squitieri, Giulia Giacomucci, Anna Poggesi, Emanuele Puca, Silvia Bianchi, Michele Ragno, Leonardo Pantoni

**Affiliations:** 1 Emergency Department, Stroke Unit, AOU Careggi, Florence, Italy; 2 NEUROFARBA Department, University of Florence, Florence, Italy; 3 IRCCS Fondazione Don Carlo Gnocchi, Florence, Italy; 4 Division of Neurology, Madonna del Soccorso Hospital, ASUR Marche, Ascoli Piceno, San Benedetto del Tronto, Italy; 5 Neurology and Neurometabolic Unit, Department of Medical Surgical and Neurological Sciences, University of Siena, Siena, Italy; 6 Pianeta Salute, Folignano, Ascoli Piceno, Italy; 7 Stroke and Dementia Lab, Department of Biomedical and Clinical Sciences, University of Milan, Milan, Italy

**Keywords:** CADASIL, Acute stroke, Thrombolysis, Intravenous alteplase

## Abstract

**Background:**

Cerebral autosomal dominant arteriopathy with subcortical infarcts and leukoencephalopathy (CADASIL) is a genetic small vessel disease responsible for recurrent ischemic strokes, often with a progressive course leading to dementia and disability. On MRI, lacunes, microbleeds, and severe white matter alterations are typical features of the disease. In case of acute stroke, because of the bleeding risk associated with the disease and the doubtful efficacy of fibrinolytic treatment in a disease with poor evidence of thrombosis, the efficacy of intravenous thrombolysis remains unproven. Nevertheless, stroke is a frequent occurrence in CADASIL patients, and clinicians not unlikely may face in the emergency room the situation of a CADASIL patient with an acute stroke within the time window for thrombolysis.

**Objective:**

We report on two CADASIL patients treated with intravenous alteplase for acute ischemic stroke, and we present a review of literature aimed to report epidemiological data, efficacy and safety of intravenous thrombolysis in CADASIL patients.

**Methods:**

We performed a systematic review of medical literature published until August 2, 2022. Case reports and series in English language reporting on CADASIL patients and acute stroke were included.

**Results:**

Both patients were treated with intravenous thrombolysis without complications and had a good clinical outcome. The systematic review identified three case reports of CADASIL patients who were treated with intravenous alteplase for acute ischemic stroke; no bleedings complications were described.

**Conclusions:**

Available data on intravenous thrombolysis in CADASIL patients are scarce but suggest that this treatment can be taken into consideration for these patients.

## Introduction

Cerebral autosomal dominant arteriopathy with subcortical infarcts and leukoencephalopathy (CADASIL) (OMIM 125310) is a genetic small vessel disease mainly affecting the brain. It is the most common monogenic cause of stroke and is clinically characterized by migraine with aura, stroke, psychiatric disturbances, acute reversible encephalopathy, and cognitive impairment [1]. On magnetic resonance imaging (MRI), CADASIL is characterized by diffuse white matter T2 hyperintensities, multiple lacunes, and microbleeds [1]. The disease is caused by a mutation of the *NOTCH3* gene, encoding for a transmembrane receptor protein, whose extracellular domain seems to be a major component of the granular osmiophilic material (GOM) [2] visible on electronic microscopy that accumulate near the vascular smooth muscle cells and represent the histological hallmark of the disease. Microscopic analysis has shown a degeneration of the vascular smooth muscle cells of the cerebral vessels, especially of the small penetrating and leptomeningeal arterioles, with consequent fibrosis and progressive thickening of the wall that lead to vascular insufficiency [3]. However, the wall thickening was not associated with a reduction of the lumen diameter on pathological studies [4], and an impaired cerebrovascular reactivity rather than a narrowing of the small vessels has been supposed as the cause of the cerebral hypoperfusion and of the development of the brain lesions [3].

Many studies aimed at finding potential therapies for the disease, considering the idea of a toxic gain of function of NOTCH3 extracellular domain, and hypothesized the elimination or the silencing of the mutated domain as well as genetic therapies; however, they are still in an experimental phase[5]. Therefore, at present, there are no specific treatments for CADASIL nor have at the moment been highlighted strategies for the secondary prevention of cerebrovascular events, and patients are treated according to guidelines for sporadic ischemic stroke. Because the mechanism of acute ischemic stroke occurring in CADASIL is also unknown, also given the increased risk of bleeding in these patients[6], the use of thrombolysis in the acute phase remains an option with unclear evidence in literature.

We report on two CADASIL patients with acute ischemic stroke treated with intravenous thrombolysis and present a literature review aimed to clarify epidemiological data, efficacy, and safety of thrombolysis in CADASIL patients.

## Methods

### Systematic literature review

Two authors (F. P. and S. T.) performed a systematic review of medical literature by searching two comprehensive medical databases, namely, PubMed and Embase, from inception to August 2, 2022.

The search query employed were “((CADASIL) OR (Cerebral Autosomal Dominant Arteriopathy with Subcortical Infarcts and Leukoencephalopathy)) AND (thrombolysis [Title/Abstract] OR fibrinolysis [Title/abstract] OR acute stroke [Title/abstract] OR acute stroke treatment [Title/abstract] OR intravenous tissue plasminogen activator [Title/abstract] OR rtPA [Title/abstract])” for Pubmed, and (CADASIL:ab,ti OR (Cerebral:ab,ti AND Autosomal:ab,it AND Dominant:ab,ti AND Arteriopathy:ab,ti AND with:ab,ti AND Subcortical:ab,ti AND Infarcts:ab,ti AND Leukoencephalopathy:ab,ti)) AND (thrombolysis:ab,ti OR fibrinolysis:ab,ti OR (acute:ab,ti AND stroke:ab,ti) OR (acute:ab,ti AND stroke:ab,ti AND treatment:ab,ti) OR (intravenous:ab,ti AND tissue:ab,ti AND plasminogen:ab,ti AND activator:ab,ti) OR rtpa:ab,ti) for Embase. Case reports and series published in English language presenting CADASIL patients treated with intravenous thrombolysis for acute stroke were included. All the abstracts were screened and selected independently by the two authors to be included in the analysis.

## Results

### Patient 1

A 65-year-old woman was admitted to the emergency room (ER) because of the sudden appearance of weakness and loss of sensation on the right side of the body. She was a heavy smoker (60 packs per year), had a history of migraine, depressive episodes, and suffered from hypercholesterolemia, but she did not assume any therapy. She was not affected by hypertension and did not take antiplatelet or anticoagulant therapies. In the past, she had suffered from spontaneous deep vein thrombosis. Her father and her paternal uncles had been affected by ischemic and hemorrhagic strokes; one sister, one brother, and a cousin in paternal line suffered from psychiatric symptoms; one sister and two nephews suffered from migraine, while there was no history of dementia.

**Fig.1:**
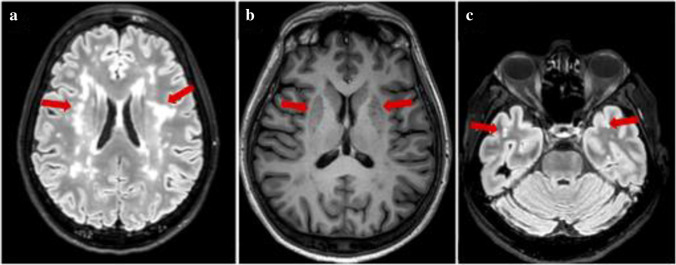
Patient 1. a Baseline FLAIR MRI axial plane showing widespread leukoencephalopathy involving external capsule (red arrows); **b** baseline T1-weighted images showing enlarged perivascular spaces in basal ganglia (red arrows); **c** 6-month follow-up FLAIR MRI revealing hyperintensities with mildly anterior temporal poles involvement (red arrows)

On admission, the neurological examination showed mild right hemiparesis and hypoesthesia. The National Institute Health Stroke Scale (NIHSS)[7] score was 3. Brain computed tomography (CT) showed frontal bilateral signs of microangiopathy with lacunes in corona radiata and multiple hypodense areas in the frontal white matter; in consideration of the patient’s symptoms, CT angiography was not performed. In absence of contraindications, 120 min after the onset of symptoms, intravenous thrombolysis with alteplase 0.9 mg/kg was started. Immediately after thrombolysis, the neurological examination was stable while improved after 24 h to a NIHSS of 1 (mild hyposthenia in the right hand and leg). Further improvement occurred on the second day, and an almost complete recovery was achieved at discharge, 10 days after onset, with minimal residual hyposthenia in the IV and V fingers of the right arm (NIHSS = 0). The brain CT performed after 24 h was unchanged, and aspirin 300 mg and atorvastatin 40 mg were started. On MRI and MR angiography, there were no areas of restricted diffusion or flow alterations of the cerebroafferent vessels, while many areas of hyperintensity on T2-weighted sequences in the subcortical white matter involving the external capsule (Fazekas scale score = 2) (Fig. 1a) and enlarged perivascular spaces without lacune or microbleeds were present (Fig. 1b). Transthoracic echocardiogram, electrocardiogram (ECG), and 24-h ECG did not show structural or arrhythmic alterations; ultrasound cervical vessels confirmed the presence of non-significant atheromatous plaques in both internal carotid arteries. Leg venous ultrasound was normal. To exclude inflammatory-immunologic diseases, such as vasculitis, the patient underwent cervico-dorsal spinal cord MRI, cerebrospinal fluid analysis, visual-evoked potentials and hematological tests for autoimmunity, coagulation, and infectious disease screening, without any pathological finding. Brain MRI was repeated after 6 months, and the lesions were unchanged in comparison with the previous exam. One month after stroke, the patient began to complain of depressive symptoms and a worsening of migraine. Brain MRI was re-evaluated, and small hyperintense areas on T2-weighted sequences were noted in the anterior poles of the temporal lobe (Fig. 1c) as well as in the external capsule. Taking also into account the family history, CADASIL scale was calculated [8] obtaining a score of 15, and the disease was suspected. The analysis of the *NOTCH3* gene was performed revealing the pathogenic mutation c.2851C > T (p.Arg 951Cys) on exon 18.

### Patient 2

A 57-year-old man presented to the ER because of the sudden occurrence of speech disturbances. He had a previous diagnosis of CADASIL (c.3094C > T on exon 19; p.Arg1006Cys) and suffered from migraine with sensitive aura; furthermore, he was affected by hyperuricemia, dyslipidemia, hyperhomocysteinemia, and hypertension. He was taking aspirin 100 mg (since the diagnosis of CADASIL), folic acid, allopurinol, and a red rice supplement for his dyslipidemia.

His mother was also affected by CADASIL and was symptomatic for epilepsy, recurrent strokes, mood disorder, and dementia. The patient was unable to report any illness of the other relatives.

**Fig. 2:**
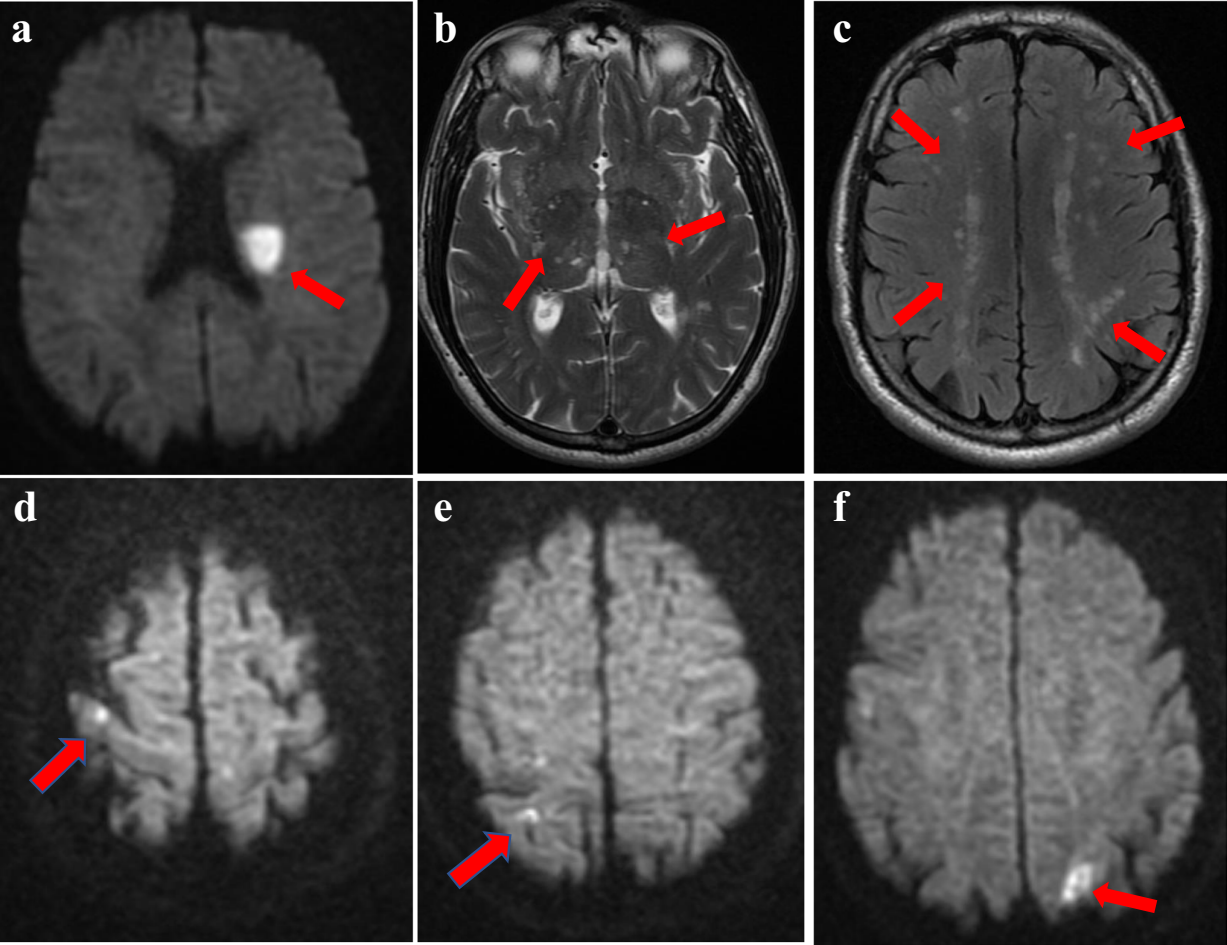
Patient 2. a–c Brain MRI performed after the first stroke. a MRI DWI image showing a restricted lesion in the left corona-radiata; **b** MRI T2-weighted image showing multiple lacunae and enlarged perivascular spaces in the basal ganglia (red arrows); **c** FLAIR MRI axial plane showing mild-to-moderate leukoencephalopathy (red arrows); **d**–**f** Brain MRI performed after the second stroke. **d**, **e** MRI DWI images showing cortical restricted lesion in the right parietal lobe; **f** MRI DWI images showing cortico-subcortical restricted lesion in the left occipital lobe

The neurological examination showed right hemiparesis and dysarthria (NIHSS = 4). Brain CT and CT angiography did not show acute lesions or vessels occlusions, and intravenous thrombolysis was started within 4.5 h of onset. Twenty-four hours after treatment, brain CT showed the appearance of a new infarct in the left the corona radiata, with no evidence of bleedings. Brain MRI performed a few days later confirmed the finding and showed multifocal periventricular and subcortical hyperintensities on T2-weigthed and FLAIR sequences (Fazekas scale score = 2) without involvement of the temporal poles and external capsule, multiple lacunae in the basal ganglia, and enlarged perivascular spaces in the basal ganglia and centrum semiovale; microbleeds were absent (Fig. 2a–c).

**Fig. 3:**
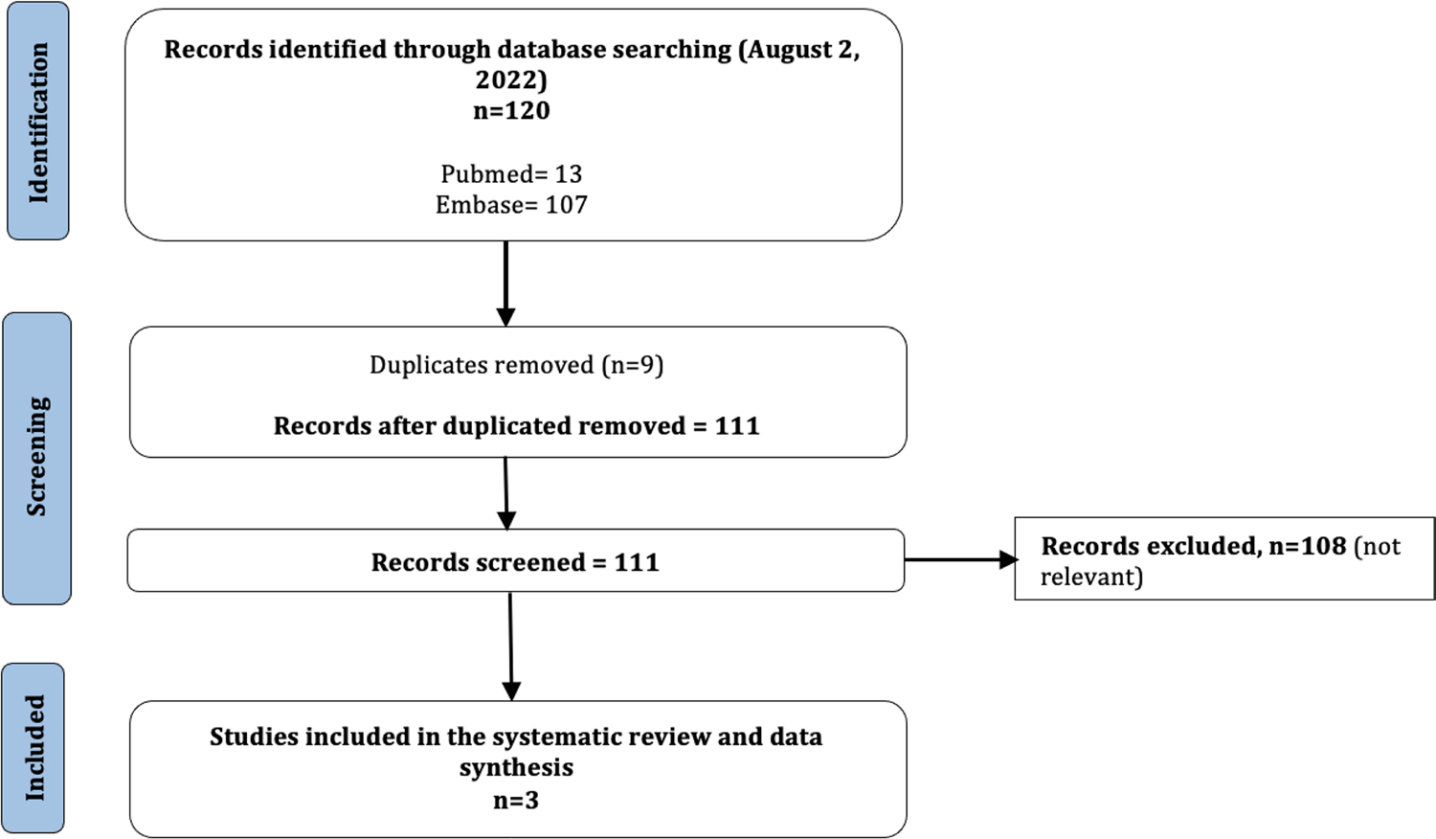
PRISMA flow diagram

The patient presented progressive improvement of the neurological deficits, and on discharge, he had only mild dysarthria (NIHSS = 1). Aspirin was substituted by clopidogrel 75 mg. Six months later, the patient presented again to the ER for the sudden appearance of severe dysarthria in the absence of other neurological deficits (NIHSS = 2). Brain CT was negative for bleedings or new ischemic lesions; intravenous thrombolysis was administered. No bleedings were found on 24-h post-treatment brain CT. Brain MRI showed multiple cortical and cortical/subcortical restricted-diffusion areas on right parietal lobe and left occipital lobe (Fig. 3). Exams were performed to investigate the pathogenesis of these acute lesions revealing patent foramen ovale (PFO) with severe right-left shunt. Neurological disturbances partially recovered but mild dysarthria on discharge (NIHSS = 1) persisted. A few weeks later, the patient was submitted to PFO closure, which was successfully completed.

## Systematic literature review

Figure 3 shows the PRISMA flow diagram. Out of 120 records detected by the search strategy, 9 were removed as duplicates. Titles and abstracts of the remaining 111 papers were screened. We excluded articles not consistent with the aim of the review (*n* = 108). Finally, we reviewed 1 paper and 2 abstracts for a total of 3 patients. Table 1 reports the demographic data, genetic mutations, and clinical and neuroimaging features that could be obtained [9–11], compared with our patients’ data. Two patients were younger and one older than our patients; all presented mild-to-moderate neurological deficits at stroke occurrence compatible with subcortical infarcts, as typical in this disease. Regarding neuroimaging, we have no data for 1 patient, the older patient presented multiple infarcts and multiple microbleeds, and the youngest severe leukoencephalopathy and no microbleeds. In two patients, new acute ischemic lesion causing the stroke was found on MRI. No patient had hemorrhagic complications after i.v. thrombolysis.

**Table 1:** Demographic data, genetic mutations, and clinical and neuroimaging features of systematic review’s patients, compared with our patients’ data. *rTPA*, recombinant tissue plasminogen activator. *TIA*, transient ischemic attack. *N/A*, not available

Systematic literature review Author, year	Gender	NOTCH3 mutation	Age at stroke occurrence	Acute stroke symptoms	Vessels occlusions	MRI findings	Stroke recovery	Bleeding after rTPA	Previous TIA/stroke	Cognitive impairment	Migraine	Psychiatric disorders
Corea et al. (2020)^9^	Male	C544T mutation (Arg182Cys)	42	Hemiparesis and severe dysarthria (NIHSS = 6)	No	DWI hyperintensity in left basal ganglia	Good improvement	No	N/A	N/A	N/A	N/A
Khan et al. (2016)^10^	Female	C406T mutation (Arg110Cys)	35	Right-sided hemiparesis, hemiparesthesia and transcortical motor aphasia (NIHSS = 8)	No	No new areas of diffusion restriction	Full	No	Previous strokes	N/A	N/A	N/A
Piccolo et al. (2021)^11^	Male	C1630T mutation (Arg544Cys)	66	Right-sided numbness and sensory ataxia	No	Bilateral lacunar acute lesions	N/A	No	Previous lacunar stroke	N/A	N/A	N/A
Case report												
*Patient 1*	Female	C2851T mutation (Arg951Cys)	65	Right hemiparesis and hypoesthesia (NIHSS = 3)	No	No new areas of diffusion restriction	Full	No	No	No	Migraine without aura	Depressive episodes
*Patient 2*	Male	C3094T mutation (Arg1006Cys)	57	Right hemiparesis and dysarthria (NIHSS = 4)	No	DWI hyperintensity in the left the corona radiata	Good improvement	No	No	No	Migraine with sensitive aura	No
				Dysarthria (NIHSS = 2)	No	Multiple cortical and cortical/subcortical restricted-diffusion areas on right parietal lobe and left occipital lobe	Good improvement	No				
Systematic literature review Author, year			Grade of leukoencephalopathy			Lacunes		Microbleeds		Hypertension		Antiplatelet/anticoagulant therapies
Corea et al. (2020)^9^			N/A			N/A		N/A		N/A		N/A
Khan et al. (2016)^10^			Severe			N/A		None		No		Aspirin 325 mg
Piccolo et al. (2021)^11^			N/A			Multiple		Multiple		No		N/A
Case report												
*Patient 1*			Moderate (Fazekas 2)			No		No		No		None
*Patient 2*			Moderate (Fazekas 2)			Multiple in the basal ganglia		No		Yes		Aspirin 100 mg

## Discussion

We report on the fourth and the fifth CADASIL patients treated with intravenous alteplase for acute ischemic stroke who showed a progressive improvement of the neurological deficits until regression over a few days and without hemorrhagic complications.

Stroke is a quite frequent event in CADASIL patients and therefore stroke clinicians not unlikely may face in the emergency room the situation of a CADASIL patient with an acute stroke within the time window for intravenous thrombolysis; anyway, nowadays, there are few reported cases of thrombolysis in CADASIL, and the reasons may be different. First of all, the pathogenesis of the ischemic events in CADASIL has not been clarified yet; as a matter of fact, the pathological findings of the disease are GOM and degeneration of vascular smooth cells, but the thrombotic genesis of the ischemic events with occlusions of small vessels has never been demonstrated [12]. However, CADASIL patients may be affected not only by an ischemic stroke due to small vessel disease, but embolic stroke may also occur as reported in patient 2. Furthermore, CADASIL has been associated with a greater risk of intracerebral bleeding, given the frequent finding of microbleeds and the possible occurrence of spontaneous cerebral hemorrhages especially in patients affected by hypertension and with extensive neuroimaging typical alterations [6, 13, 14]. Anyway, the occurrence of intracerebral hemorrhage remains uncommon, although its actual frequency is not known [14]. It remains to understand if the intracerebral bleeding, mostly involving typical brain areas, is due to risk factors such as hypertension or antiplatelet/anticoagulant therapies or to CADASIL vasculopathy itself [14]. Regarding hemorrhagic risk after thrombolysis in CADASIL, available information is insufficient to draw conclusions. Among the reported patients (including ours), no bleedings occurred. Two patients were on aspirin, and one of them suffered also from hypertension; four had brain lesions on MRI but microbleeds were present only in one.

The 2020 Consensus recommendations of the European Academy of Neurology on the treatment of monogenic stroke state that thrombolysis is not recommended for lacunar stroke in CADASIL patients [15]. However, according to the new European Stroke Organisation (ESO) guidelines on intravenous thrombolysis for acute ischemic stroke, there is no strong evidence that intravenous thrombolysis should be avoided in case of mild strokes [16]; CADASIL is not mentioned in these guidelines. Moreover, CADASIL is still an underdiagnosed disease, and some patients with acute stroke treated with thrombolysis could have been affected by the disease without knowing the diagnosis.

In conclusion, we reported on two patients and a literature review that revealed no bleeding complications and good clinical outcome in CADASIL patients treated with intravenous alteplase for acute ischemic stroke. These results suggest that thrombolysis could be taken into account in this disease independently from the ischemic stroke etiology.

## References

[1] Chabriat H, Joutel A, Dichgans M, Tournier-Lasserve E, Bousser M (2009). CADASIL. Lancet Neurol.

[2] Ishiko A, Shimizu A, Nagata E, Takahashi K, Tabira T, Suzuki N (2006). Notch3 ectodomain is a major component of granular osmiophilic material (GOM) in CADASIL. Acta Neuropathol.

[3] Locatelli M, Padovani A, Pezzini A (2020). Pathophysiological mechanisms and potential therapeutic targets in cerebral autosomal dominant arteriopathy with subcortical infarcts and leukoencephalopathy (CADASIL). Front Pharmacol.

[4] Dong H, Ding H, Young K, Blaivas M, Christensen PJ, Wang MM (2013). Advanced intimal hyperplasia without luminal narrowing of leptomeningeal arteries in CADASIL. Stroke.

[5] Bersano A, Bedini G, Oskam J, Mariotti C, Taroni F, Baratta S, Parati EA (2017). CADASIL: treatment and management options. Curr Treat Options Neurol.

[6] Rinnoci V, Nannucci S, Valenti R, Donnini I, Bianchi S, Pescini F, Dotti MT, Federico A, Inzitari D, Pantoni L (2013). Cerebral hemorrhages in CADASIL: report of four cases and a brief review. J Neurol Sci.

[7] Kwah LK, Diong J (2014). National Institutes of Health Stroke Scale (NIHSS). J Physiother.

[8] Pescini F, Nannucci S, Bertaccini B, Salvadori E, Bianchi S, Ragno M, Sarti C, Valenti R, Zicari E, Moretti M, Chiti S, Stromillo ML, De Stefano N, Dotti MT, Federico A, Inzitari D, Pantoni L (2012). The Cerebral Autosomal-Dominant Arteriopathy With Subcortical Infarcts and Leukoencephalopathy (CADASIL) Scale: a screening tool to select patients for NOTCH3 gene analysis. Stroke.

[9] Corea F, Acciaresi M, Micheli S, Guidubaldi A, Carducci D, Brustenghi P, mZampolini M (2020). Use of intravenous tissue plasminogen activator for a patient with cerebral autosomal dominant arteriopathy with subcortical infarcts and leukoencephalopathy (CADASIL). Int J Stroke.

[10] Khan MT, Murray A, Smith M (2016). Successful use of intravenous tissue plasminogen activator as treatment for a patient with cerebral autosomal dominant arteriopathy with subcortical infarcts and leukoencephalopathy: a case report and review of literature. J Stroke Cerebrovasc Dis.

[11] Piccolo L, Pensato U, Forlivesi S, Gentile M, Merli E, Naldi F, Cirillo L, Cianci F, Zini A (2021). Large-vessels vasculitis associated with a rare Cadasil genotype : a double pathology of cerebral arteries. Eur Stroke J.

[12] Di Donato I, Bianchi S, De Stefano N, Dichgans M, Dotti MT, Duering M, Jouvent E, Korczyn AD, Lesnik-Oberstein SA, Malandrini A, Markus HS, Pantoni L, Penco S, Rufa A, Sinanović O, Stojanov D, Federico A (2017). Cerebral Autosomal Dominant Arteriopathy with Subcortical Infarcts and Leukoencephalopathy (CADASIL) as a model of small vessel disease: update on clinical, diagnostic, and management aspects. BMC Med.

[13] Nannucci S, Rinnoci V, Pracucci G, MacKinnon AD, Pescini F, Adib-Samii P, Bianchi S, Dotti MT, Federico A, Inzitari D, Markus HS, Pantoni L (2018). Location, number and factors associated with cerebral microbleeds in an Italian-British cohort of CADASIL patients. PLoS ONE.

[14] Palazzo P, Le Guyader G, Neau JP (2021). Intracerebral hemorrhage in CADASIL. Rev Neurol (Paris).

[15] Mancuso M, Arnold M, Bersano A, Burlina A, Chabriat H, Debette S, Enzinger C, Federico A, Filla A, Finsterer J, Hunt D, Lesnik Oberstein S, Tournier-Lasserve E, Markus HS (2020). Monogenic cerebral small-vessel diseases: diagnosis and therapy. Consensus recommendations of the European Academy of Neurology. Eur J Neurol.

[16] Berge E, Whiteley W, Audebert H, De Marchis GM, Fonseca AC, Padiglioni C, de la Ossa NP, Strbian D, Tsivgoulis G, Turc G (2021) European Stroke Organisation (ESO) guidelines on intravenous thrombolysis for acute ischaemic stroke. Eur Stroke J 6:I-LXII. 10.1177/2396987321989865 10.1177/2396987321989865 PMC7995316 33817340

